# Laser-induced CNV following hair removal procedure

**Published:** 2019

**Authors:** Ioana Teodora Tofolean, Radgonde Amer

**Affiliations:** * Eye Emergency Hospital, Bucharest, Romania; ** Department of Ophthalmology, Hadassah Medical Center, Jerusalem, Israel

**Keywords:** hair removal, diode laser, choroidal neovascularization, OCTA

## Abstract

**Objective:** To describe the chronological features of choroidal neovascular membrane (CNV) development subsequent to accidental firing of diode laser into the eye of a young female during hair epilation.

**Methods:** Descriptive case report.

**Results:** The patient presented one week after the laser injury to a local ophthalmologist complaining of RE (right eye) blurred central vision. Snellen’s visual acuity (VA) was 6/ 7.5. Optical coherence tomography (OCT) showed focal disruption of the ellipsoid and the interdigitation zones. Four weeks later, she presented with worsening symptoms and RE VA 6/ 15. Funduscopy revealed a perifoveal grayish lesion with adjacent retinal hemorrhage, which, on fluorescein angiography, was leaking, compatible with CNV. OCT showed a dome-shaped sub-retinal pigment epithelium lesion with extension into the subretinal space and little subretinal fluid. The patient was treated with one intravitreal bevacizumab injection. There was rapid regression of the CNV and improvement of VA to 6/ 7.5 at one-month visit and to 6/ 6 at 6-month visit.

**Conclusion:** All the laser procedures should be conducted with intensive care for both the patient and the laser surgeon since inadvertent effects are constantly being reported due to lack of adherence to safety measures.

## Introduction

Laser or light-based hair removal is one of the most commonly used medical procedures nowadays, with increasing addressability to our society. Although the information about the lasers’ ability to nonspecifically damage hair follicles dates to over 50 years ago, the first successful laser for long-term hair removal was proposed in the late ‘90s, after confirming the selective photothermolysis theory [ **1**].

Melanin functions as a target chromophore for wavelengths in the red and near infrared portion of the electromagnetic spectrum, but in order to achieve permanent hair depletion, heat needs to diffuse from the “light” target towards the “biological” target, namely the follicular stem cells [ **2**]. Depending on the photons’ wavelength, hair removal is most commonly achieved by red spectrum ruby (694 nm), alexandrite laser (755 nm), diode laser (700-1000 nm), neodymium-doped yttrium aluminum garnet laser (Nd:YAG laser, 1064 nm), as well as by intense pulsed light devices (IPL, 590-1200 nm) [ **3**]. Beside wavelength, other factors important both for therapeutic and safety purposes include fluence (J/ cm2), pulse duration (ms), spot size (mm), adjuvant skin cooling systems and the Fitzpatrick skin phototypes.

## Methods

The present report describes the case of a young healthy female patient who developed choroidal neovascular membrane (CNV) induced by accidental firing of diode laser into her right eye (RE) during hair removal procedure while not wearing appropriate eye protection.

## Results

**Fig. 1:**
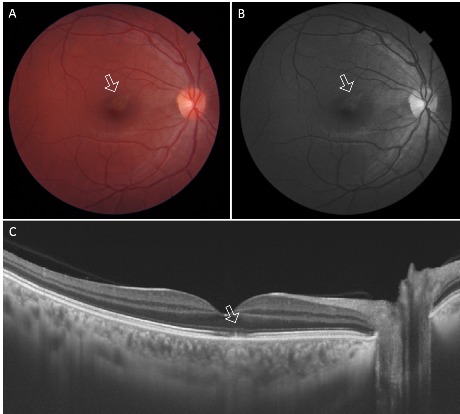
**A.** Fundus photograph of the right eye at presentation (one week after the laser injury) showing a wedge-shaped hypopigmented lesion superior to fovea with well-demarcated lighter borders (arrow). **B.** Red-free image of the right eye showing the hypopigmented perifoveal lesion with perilesional halo (arrow). **C.** Spectral-domain optical coherence tomographic image of the right fundus showing focal disruption of the ellipsoid and interdigitation zones (arrow)

A 31-year-old female without any significant past medical history underwent diode laser-assisted hair removal of the lower limbs. During the procedure, she accidentally directed the probe towards her face and while not using protective eyewear, the laser beam was fired directly into her right eye. Seven days later, the patient started to complain of blurred central vision. On examination by a local ophthalmologist, Snellen’s visual acuity (VA) was 6/ 7.5 in RE and 6/ 6 in the left eye (LE). The intraocular pressures and the anterior segments were normal in both eyes. Funduscopy revealed a wedge-shaped perifoveal hypopigmented lesion, superior to fovea, in RE ( **Fig. 1**). Spectral-domain optical coherence tomography (OCT) showed a focal disruption of the ellipsoid and interdigitation zones ( **Fig. 1**). LE fundus exam was normal.

**Fig. 2:**
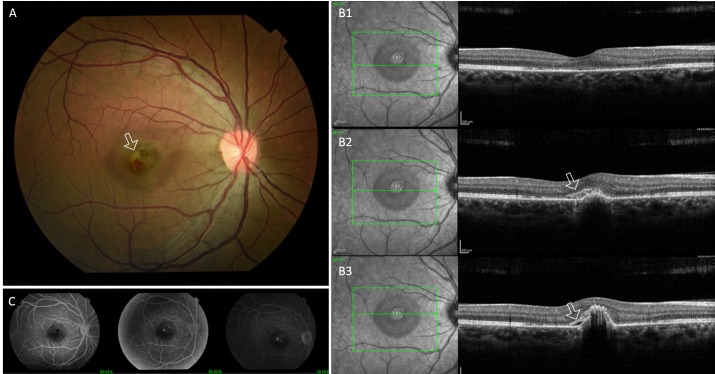
**A.** Fundus photograph five weeks after the laser injury showing perifoveal grayish lesion with adjacent retinal hemorrhage (arrow). **B.** Macular optical coherence tomography with horizontal scans of the fovea and of the perifoveolar area. B1. Disruption of the outer retinal bands is noted in the foveal area (external limiting membrane, ellipsoid zone, interdigitation zone, and retinal pigment epithelium). B2. An elevated dome-shaped lesion is noted in the sub-retinal pigment epithelium with an extension to the subretinal space and a hyperreflective lesion in the area of the retinal hemorrhage (arrow). B3. Additional small amount of subretinal fluid is noted (arrow). **C.** Fluorescein angiography demonstrating an actively leaking choroidal neovascular membrane

**Fig. 3:**
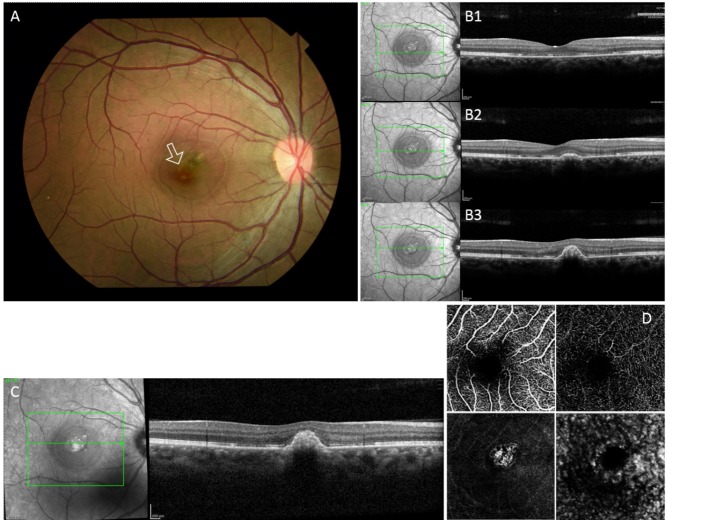
**A.** Fundus photograph of the right eye at one month after the intravitreal bevacizumab showing a small, pigmented perifoveal lesion with a smaller retinal hemorrhage. **B.** Macular optical coherence tomography of the right eye showing in B1: disruption of the outer retinal bands, in B2: small elevation of the retinal pigment epithelium and in B3: resolution of the subretinal hyperreflective lesion and the subretinal fluid that were noted in Fig. 2. **C.** Macular optical coherence tomography of the right eye at two months after the intravitreal bevacizumab showing a dome-shaped lesion with resolution of the hyperreflective dots that were seen on its apex in Fig. 2. **D.** Macular optical coherence tomography angiography of the right eye showing a regressed, choroidal neovascular membrane (well-defined lesion without significant loops or dense branching)

Five weeks after the initial trauma, she presented because of worsening of the previously mentioned complaints with an enlarging central scotoma. RE VA was 6/ 15. Funduscopy revealed a perifoveal grayish lesion with adjacent retinal hemorrhage ( **Fig. 2**) which on fluorescein angiography was leaking, compatible with CNV ( **Fig. 2**). OCT showed a dome-shaped sub-retinal pigment epithelium lesion with extension into the subretinal space and a hyperreflective subretinal lesion compatible with the retinal hemorrhage observed on funduscopy and an additional small amount of subretinal fluid ( **Fig. 2**). The patient was treated with one intravitreal bevacizumab injection (1.25mg/ 0.05cc). There was rapid regression of the CNV and improvement of VA to 6/ 7.5 at one-month-follow up, which remained stable at two-months of follow-up with total resolution of retinal hemorrhage ( **Fig. 3**). Optical coherence tomography angiography (OCTA) showed a regressed CNV with no active branching pattern ( **Fig. 3**). Six-months later, VA was 6/ 6 with no recurrence of CNV.

## Discussion

We presented the case of a healthy young female who developed CNV after sustaining diode laser-induced macular injury because of the insecure use of laser during hair epilation. We demonstrated the chronological features of CNV development with initial damage to the outer retinal bands specifically the ellipsoid and the interdigitation zones, with subsequent formation of CNV associated with subretinal hemorrhage and subretinal fluid. There was prompt resolution of the CNV with anti-vascular endothelial growth factor therapy and a single injection controlled the CNV with no signs of reactivation at two months following the intravitreal injection. The laser parameters used for hair epilation in this case were unknown, but most currently used lasers operate at high emission levels and therefore, are designated in the highest hazard classes (class 3B and class 4). 

Direct exposure to radiation represents a hazard to unprotected eyes. A wide range of ocular adverse effects following laser procedures have been reported worldwide, through photomechanical, photothermal or photochemical mechanisms [ **4**]. Although more than one effect might induce a particular injury, short wavelength lasers produce photocoagulation (the increase of the retinal temperatures to approximately 60˚C generates protein denaturation), while long wavelengths might affect the ocular tissue through either photothermal and/ or photomechanical damage (the explosive acoustic shock shears, fragments and perforates the tissue) [ **4**].

Diode lasers for epilation may generate all the relevant wavelengths for hair removal (694 nm, 755 nm, 800/ 810 nm, 980 nm, 1064 nm) and next-generation lasers will be able to simultaneously apply multiple wavelengths [ **5**]. The most common ocular adverse effects induced by diode lasers involve the iris, encompassing anterior uveitis and iris damage followed by iris atrophy and/ or pupillary distortion [ **6**]. Lens involvement includes anterior subcapsular and nuclear cataract. The retina, which is responsible for converting electromagnetic radiation into an electric signal, is particularly vulnerable to wavelengths in the visible to near-infrared spectrum (400nm–1400nm), known as the retinal hazard region [ **6**]. Vitreous hemorrhage, retinal burns with edema, chorioretinal scarring, foveal granularity, pigment clumping, subretinal hemorrhage with consecutive photoreceptors’ damage and macular holes have all been described, either secondary to facial laser procedures (hair removal or skin treatments) or to accidental exposure to industrial and military lasers [ **6**, **7**]. Although the development of an experimental animal model of laser-induced subretinal neovascularization for macular degeneration dates back to the late ‘70s, this is, to our knowledge, the first paper describing CNV formation secondary to diode laser injury in humans. There are very few other case reports of other types of laser-induced CNV formation following hair removal procedures, most of them showing complete and constant fluid resolution at one-month follow-up after one single intravitreal bevacizumab injection [ **8**, **9**]. It seems that lesions with Bruch’s membrane damage potentially lead to CNV and the endpoint best-corrected VA depends on the magnitude of the scar formation in the fovea.

OCTA has recently emerged as a noninvasive imaging modality, depicting a real potential of replacing FA/ ICG to assess CNV in OCT imaging. Being a noninvasive substitute for FA/ ICG, but providing comparable functional information to the one obtained from dye-based angiography [ **10**], it can clearly depict features of CNV activity versus regression.

This report outlines a new case of laser-induced CNV caused by diode laser that was used in hair epilation, in the absence of any type of ocular protection. Misdirection of the laser beam towards the eye has visual-threatening consequences. Proper eye protection is critical for both the patient and the laser surgeon. Manufacturers recommend the use of specific goggles (depending on the device’s wavelengths), gauze sponges under opaque goggles to ensure eye closure or even corneal shields for interventions in the periocular area.

**Disclosures**

None. 
